# Elevated PLAUR is observed in the airway epithelium of asthma patients and blocking improves barrier integrity

**DOI:** 10.1002/clt2.12293

**Published:** 2023-10-01

**Authors:** Michael A. Portelli, Sangita Bhaker, Vincent Pang, David O. Bates, Simon R. Johnson, Andrew P. Mazar, Dominick Shaw, Christopher Brightling, Ian Sayers

**Affiliations:** ^1^ Centre for Respiratory Research NIHR Respiratory Biomedical Research Centre School of Medicine Biodiscovery Institute University Park University of Nottingham Nottingham UK; ^2^ Tumour Vascular Biology Group Biodiscovery Institute University Park University of Nottingham Nottingham UK; ^3^ Department of Pharmacology Feinberg School of Medicine Northwestern University Chicago Illinois USA; ^4^ Department of Respiratory Medicine University of Leicester University Hospitals of Leicester NHS Trust Leicester UK

**Keywords:** asthma, barrier, bronchial epithelial cells, urokinase plasminogen receptor

## Abstract

**Background:**

Expression of the urokinase plasminogen activator receptor (uPAR) is elevated in the airway epithelium in asthma; however, the contribution of uPAR to asthma pathogenesis and scope for therapeutic targeting remains unknown.

**Objectives:**

To determine (i) the expression profile of uPAR in cultured human bronchial epithelial cells (HBEC) from asthma patients, (ii) the relationship between uPAR and the epithelial barrier, including blocking uPAR functions and (iii) the function of different uPAR isoforms.

**Methods:**

uPAR levels in HBECs isolated from asthma patients and cells at air liquid interface (ALI) during differentiation were quantified. Transepithelial electrical resistance or electrical cell impedance sensing was used to relate uPAR levels to barrier properties, including effects of uPAR blocking antibodies. The functional effects of gain of function was determined using transcriptomics, in cells over‐expressing membrane (muPAR), soluble cleaved (scuPAR) or soluble spliced (ssuPAR) isoforms.

**Results:**

Elevated expression of uPAR was a feature of cultured HBECs from asthma patients, suggesting intrinsic alterations in asthma patient cells. Soluble uPAR levels inversely correlated with barrier properties of the HBEC layer in 2D and ALI. Blocking uPAR‐integrin interactions enhanced barrier formation. The gain of function cells showed limited transcriptomic changes.

**Conclusion:**

This study provides a significant advance in our understanding of the relationship between asthma, uPAR and the epithelial barrier, where elevated circulating uPAR results in a reduced cell barrier, a phenotype prevalent in asthma.

## 1 INTRODUCTION

Asthma is a common, complex, heterogeneous disease that results from the interaction between genetic and environmental factors. Asthma is characterized by airway hyper‐responsiveness, inflammation, and variable airflow obstruction. The uPAR is activated by urokinase plasminogen activator (uPA) from the 431 amino acid pro‐uPA (UniProt P00749), triggering the plasminogen/plasmin activation cycle and its extracellular proteolytic cascade.^1^ However, uPAR also exhibits functionality through non‐proteolytic activity via interaction with a number of different cell‐bound factors, such as integrins and G‐protein–coupled receptors. This allows uPAR to stimulate signaling cascades and regulate diverse functions, including cytoskeletal dynamics, cellular adhesion, and cellular migration.^1^, ^2^ These proteolytic and non‐proteolytic functions allow uPAR to play an active role in a number of pro‐airway remodeling processes, such as extracellular matrix remodeling, cell migration, coagulation, cellular proliferation, release of inflammatory cytokines, and growth factor activation.^3^, ^4^, ^5^

Altered uPAR expression and activity is linked to asthma,^4^, ^6^, ^7^, ^8^, ^9^ with elevated uPAR observed in the airway epithelium of asthma patients^4^ and correlated with epithelial proliferation in vivo.^8^ uPAR gene (*PLAUR*) polymorphisms are associated with asthma development risk and lung function decline, implicating a role in patient airway remodeling.^6^ Full‐length, membrane‐bound uPAR (muPAR) exists as a glycosylphosphatidylinositol (GPI)‐anchored protein consisting of three globular domains DI (exons 2 and 3), DII (exons 4 and 5), and DIII (exons 6 and 7).^10^ Each domain plays an important signaling role, with DI and DII involved in uPA binding, and DII and DIII involved in integrin binding (e.g., α5β1). Different soluble uPAR isoforms have also been described, include soluble cleaved (scuPAR) and soluble spliced (ssuPAR) forms.^10^, ^11^ scuPAR arises from proteolytic, glycolytic and lipolytic cleavage of or near the GPI anchor via a number of molecules, including phospholipase‐D, cathepsin‐G, MMPs and GDE3.^12^ The alternate soluble receptor arises from RNA splicing altering the *PLAUR* terminal exon, altering the amino acid sequence from position 254 with the resulting protein being unable to form the GPI anchor.

We have shown that elevated uPAR is a feature of damaged airway epithelium and regulates repair.^4^ Interestingly, elevated serum scuPAR was a feature of more severe, non‐allergic asthma patients, suggesting that specific uPAR isoforms may be more relevant to specific asthma subsets.^13^ Artificially elevating uPAR in primary airway epithelial cells in vitro generates a pro‐remodeling phenotype with overlapping and specific biological effects of isoforms on proliferation, migration, and matrix‐metalloproteinase production.^14^

Therefore, uPAR represents an appealing therapeutic target for asthma through links to altered expression and activity in the airway epithelium, where blocking selected functions of uPAR may be beneficial in halting/reversing airway remodeling changes observed. However, prior to effectively targeting uPAR, a greater understanding of (i) the potential differential expression of uPAR isoforms in asthma patient cells, (ii) the role of uPAR in epithelial cell homeostasis including barrier properties, (iii) the potential to target selected uPAR functions and (iv) the molecular mechanisms underlying the differential effects of uPAR isoforms on epithelial cell functions, is warranted.

## 2 MATERIALS AND METHODS

For additional methods see Supplementary Material S1.

### 2.1 Subject recruitment and bronchial epithelial cell isolation

Moderate to severe asthma patients and healthy control subjects were recruited from Nottingham University Hospitals (BTS step 3–5 (BTS/SIGN, 2016)) and Glenfield Hospital, Leicester, UK (Global Initiative for Asthma (GINA)) guidelines. This study was conducted in accordance with the amended Declaration of Helsinki. The Leicestershire, Rutland, and Northamptonshire ethics committee (ethics reference 4977/project approval number 6347) approved the protocol, and written informed consent was obtained from all patients. Participants from Nottingham were recruited (Ethics reference 11/EM/0062 and 12/EM/0059). Human bronchial epithelial cells (HBEC) from bronchial brushings from asthma patients (*n* = 35) and healthy controls (*n* = 19) were collected via bronchoscopy as described, with a sub‐set (*n* = 33 asthma; *n* = 18 controls) used for transcriptomic analysis (Table S1).^15^, ^16^

### 2.2 Bronchial epithelial cell culture at air liquid interface

Cell cultures from control subjects were maintained in Bronchial Epithelial Cell Growth Medium (BEGM^TM^, Lonza, UK) as previously described.^15^ For air liquid interface (ALI) cultures we used our previously established protocol^15^, ^17^ with the following change; wells were coated with 200 μL PureCol Type 1 Bovine Collagen Solution (Advanced Biomatrix, San Diego, US) diluted 1 in 100 in sterile water and incubated at 37°C for one hour before washing 3x with BEGM. Air liquid interface cultures were maintained for 21 days, supernatants were collected at days 7, 14 and 21.

### 2.3 Transepithelial electrical resistance measurements (TEER)

200 μL and 500 μL of fresh media were added to the apical and basolateral compartments. Cultures from control subjects were equilibrated at 37°C for 30 min before the measurement of transepithelial electrical resistance (TEER) using the EVOM2 epithelial Volt‐ohmmeter as per manufacturer's instructions (World Precision Instruments, UK).

### 2.4 Electrical Cell Impedance Sensing (ECIS)

Electrical cell impedance sensing (ECIS) was completed as described previously.^18^ For blocking experiments, anti‐uPAR R3 (binds to Domain 1), R4 (binds to Domain 3), ATN‐658 (binds Domains 2 and 3) or IgG1 isotype control (all 1 mg/mL) were included.^19^, ^20^, ^21^

### 2.5 Genetically engineered HBEC to over‐express membrane and soluble isoforms of uPAR

HBECs were transfected with overexpression plasmids (pcDNA3™) containing three uPAR variants: pcDNA3‐soluble cleaved (scuPAR), pcDNA3‐soluble‐spliced (ssuPAR), pcDNA3‐membrane (muPAR) and pcDNA3‐empty vector control. pcDNA3‐muPAR, pcDNA3‐ssuPAR and pcDNA3‐scuPAR constructs were generated previously (Figure S3).^10^ Transient transfections of HBEC were carried out using FuGENE® 6. HBECs were seeded at passage 4 into 6‐well plates, and transfections were carried out according to the manufacturer's instructions. Briefly, FuGENE®6 was diluted in BEGM, followed by a 5 min incubation. Plasmid DNA was then added at an experiment specific ratio, allowed to complex with the FuGENE® 6 for 15 min and then added to the cells. HBECs were harvested for RNA 24 h post‐transfection. Total RNA extraction was carried out using the Qiagen RNeasy® Mini Kit, as per the manufacturer's instructions.

### 2.6 LUMINEX measurements of proteins

Magnetic Luminex assays were performed according to the manufacturer's recommendations using a custom Human Premixed Multi‐Analyte Kit (R&D systems).

### 2.7 Statistical analyses

For cell‐based outcomes, a Mann‐Whitney test was used to determine differences between conditions/cell lines. A Kruskal‐Wallis test was performed for multiple group comparisons. Spearman's test was used to investigate correlations. *p* < 0.05 was considered significant.

## 3 RESULTS

### 3.1 *PLAUR* mRNA is up‐regulated in cultured bronchial epithelial cells isolated from patients with asthma

**FIGURE 1:**
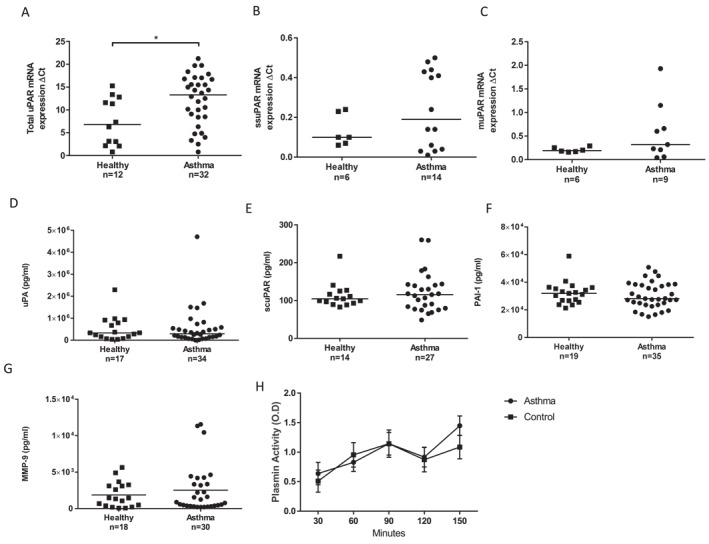
Total Urokinase plasminogen receptor mRNA levels are elevated in bronchial epithelial cells isolated from asthma patients cultured to Passage 3. (A) Total urokinase plasminogen activator receptor (uPAR) mRNA levels are significantly elevated in cultured bronchial epithelial cells from asthma patients (*n* = 32) compared to cells from healthy subjects (*n* = 12) (*p* = 0.011). (B) ssuPAR and (C) muPAR mRNA expression was not detected in all subjects and overall is not elevated in asthma patient cells (ssuPAR *n* = 6 v *n* = 14 *p* = 0.53; muPAR levels *n* = 6 v *n* = 9, *p* = 0.22). (D) scuPAR, (E) uPA, (F) PAI‐1 and (G) MMP‐9 protein expression in supernatants did not differ between groups. Median and individual subject data are shown. (H) Plasmin levels (mean+/−SEM) were not different between asthma (*n* = 5) and control (*n* = 16) cell supernatants. *p < 0.05.

To address whether observed uPAR up‐regulation in the asthma patient biopsy epithelium is due to the inflammatory environment or a feature of the epithelium per se, we determined the mRNA expression profile driven by total uPAR and uPAR variants generated by splicing in bronchial epithelial cells isolated from control subjects and asthma patients and cultured in vitro. Asthma patients that underwent bronchoscopy were predominantly moderate‐severe (GINA) and showed a female dominance (Table S1). All isolated and cultured cells demonstrated epithelial cell morphology and expressed epithelial marker cytokeratin 14 (CK‐14) and E‐cadherin. Total uPAR, membrane (m)uPAR and soluble splice (ss)uPAR mRNA expression was determined (Figure 1A–C). Total uPAR mRNA levels were significantly elevated in asthma patient cells cultured in vitro compared with healthy subject cells (Figure 1A: median ‐ control = 6.82 CI (4.14–10.76), case = 13.31 CI (10.14–14.18), *p* = 0.011). However, muPAR and ssuPAR mRNA showed no significant difference although the trend to elevated expression in cells cultured from asthma patients was apparent (Figure 1B,C). At the protein level, secreted scuPAR, uPA, plasminogen activator inhibitor‐1 (PAI‐1) and matrix metalloprotease‐9 (MMP‐9; as a marker of pathway activity) were measured; however, there was no significant difference in expression between groups (Figure 1D–G). These uPA‐uPAR pathway components showed modest correlated expression with suPAR protein levels (uPA *r* = 0.40 (*p* = 0.02), PAI‐1 *r* = 0.41 (*p* = 0.016) MMP‐9 *r* = 0.37 (*p* = 0.04)) in cells from asthma patients not from control subject cells (Figure S1). There was no difference in plasmin activity between cases and control cell supernatants (Figure 1H).

### 3.2 Soluble cleaved uPAR (scuPAR) is differentially expressed during bronchial epithelial cell differentiation and correlates with barrier properties in air liquid interface cultures

**FIGURE 2:**
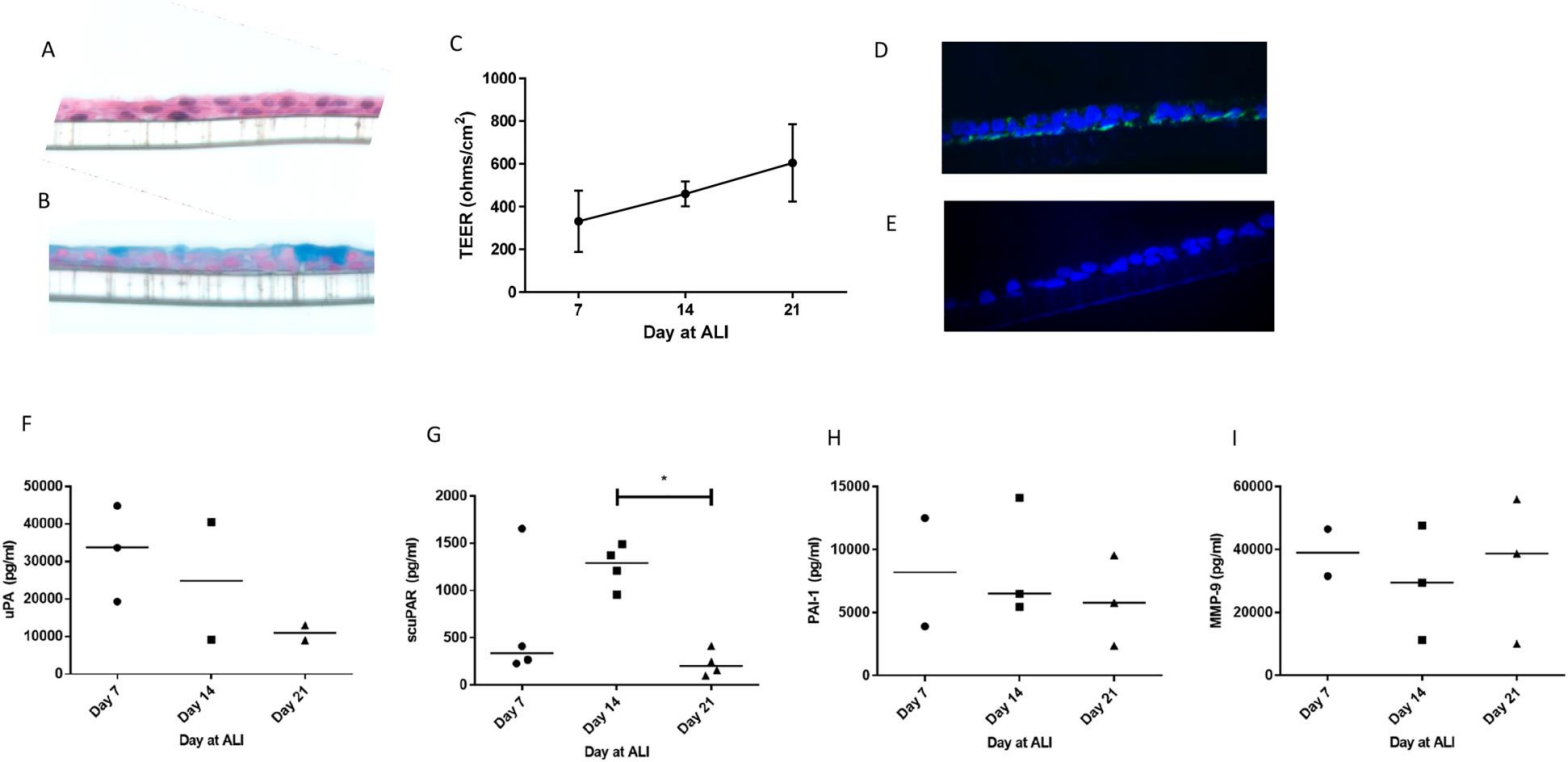
Urokinase plasminogen receptor protein expression levels in differentiating primary bronchial epithelial cells. Passage 3 HBECs were grown at air liquid interface (ALI) and on day 21 were collected, fixed and stained with (A) hematoxylin and eosin or (B) Alcian blue (representative images). During these differentiation experiments (C) transepithelial electrical resistance (TEER) developed (mean ± SEM). Cell layers show staining for (D) urokinase plasminogen activator receptor (uPAR) (green) and e) IgG1 isotype with DAPI (blue) (representative images) (x40 objective). Basolateral supernatants were harvested on days 7, 14 and 21 and (F) uPA, (G) suPAR, (H) PAI‐1 and (I) MMP‐9 protein (pg/ml) were measured using Luminex (*n* = 4 independent experiments, mean ± SEM).

Previously, we have reported that aberrant expression and regulation of uPAR and particularly, scuPAR may have implications on airway structural changes and epithelial function.^14^ However, these and our earlier work were focused on submerged 2D cultures of HBECs that may not fully address the complexity of the airway epithelial layer in the lung. To address this we therefore completed a series of experiments using the more physiologically relevant ALI model. These bronchial epithelial cell layers show a pseudo‐stratified structure (Figure 2A) and stain for mucins (Figure 2B). These cell cultures differentiate as observed morphologically and by their TEER profile (Figure 2C). The total uPAR expression was confirmed by immunofluorescence on day 21, showing expression mainly in basal cells (Figure 2D,E). To begin to understand the contribution of the uPA‐uPAR pathway during differentiation , we measured uPA, scuPAR, PAI‐1 and MMP‐9 protein levels in basolateral supernatants across the differentiation period (Figure 2F–I). These data showed that scuPAR was elevated on day 14 compared with day 21, while other protein levels were not different over time.

**FIGURE 3:**
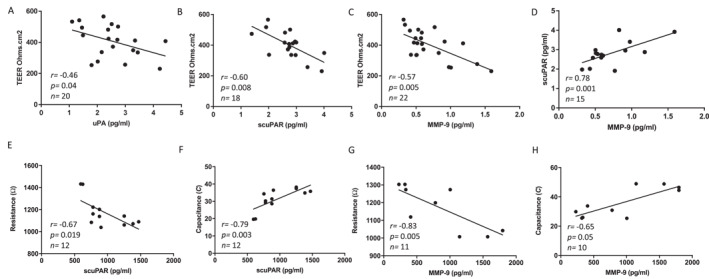
In air liquid interface (ALI) and submerged cultures of primary bronchial epithelial cells, soluble cleaved urokinase plasminogen activator receptor (uPAR) and MMP‐9 levels negatively correlate with epithelial barrier integrity. On day 14 ALI, (A) scuPAR, (B) uPA, and (C) MMP‐9 protein expression (pg/ml) negatively correlated with transepithelial electrical resistance (TEER) (Ω.cm2); scuPAR; uPA, MMP‐9. (D) MMP‐9 and scuPAR levels were positively correlated, suggesting coordinated expression (NHBEC donor 3 where *n* = individual data points. Spearman's rank correlation). Representative of *n* = 2‐4 donors. Resistance and capacitance in submerged cells were measured using Electrical cell impedance sensing (ECIS) over 72 h. In submerged cultures (E‐H), scuPAR and MMP‐9 levels in supernatants inversely correlated with resistance and positively correlated with capacitance. All experiments (*n* = 2‐4 donors).

To relate protein levels to the barrier integrity we compared levels of these proteins to TEER measurements on day 14, these data demonstrated several significant correlations notably; scuPAR (*p* = 0.008); uPA (*p* = 0.04) and MMP‐9 (*p* = 0.005) protein levels negatively correlated with barrier integrity (i.e. Transepithelial electrical resistance, Figure 3A–C). scuPAR levels also correlated with MMP‐9 levels (Figure 3D). Interestingly, in the fully differentiated layer (day 21) these relationships were not present (Figure S2), suggesting the uPA‐uPAR pathway is particularly important in establishing a barrier, that is, during repair/regeneration as our earlier data suggested.^4^

### 3.3 Soluble cleaved uPAR (scuPAR) is negatively correlated with epithelial barrier integrity and positively correlated with cell adhesion and spreading in submerged culture

To relate back to our initial experiments in 2D culture, we completed an analogous series of barrier experiments using submerged cultured cells in combination with ECIS. This independently confirmed the negative correlation between scuPAR and MMP‐9 protein levels with epithelial barrier formation, albeit this time measured via resistance (Figure 3E,G). In these experiments, we additionally observed a positive correlation between scuPAR and MMP‐9 protein levels and capacitance, which measures cell adhesion and spreading, again suggesting a role for the uPA‐uPAR pathway in the epithelial cell homeostasis and barrier development (Figure 3F,H).

### 3.4 Targeting uPAR using therapeutic monoclonal antibodies can modify epithelial cell homeostasis and promote barrier formation

**FIGURE 4:**
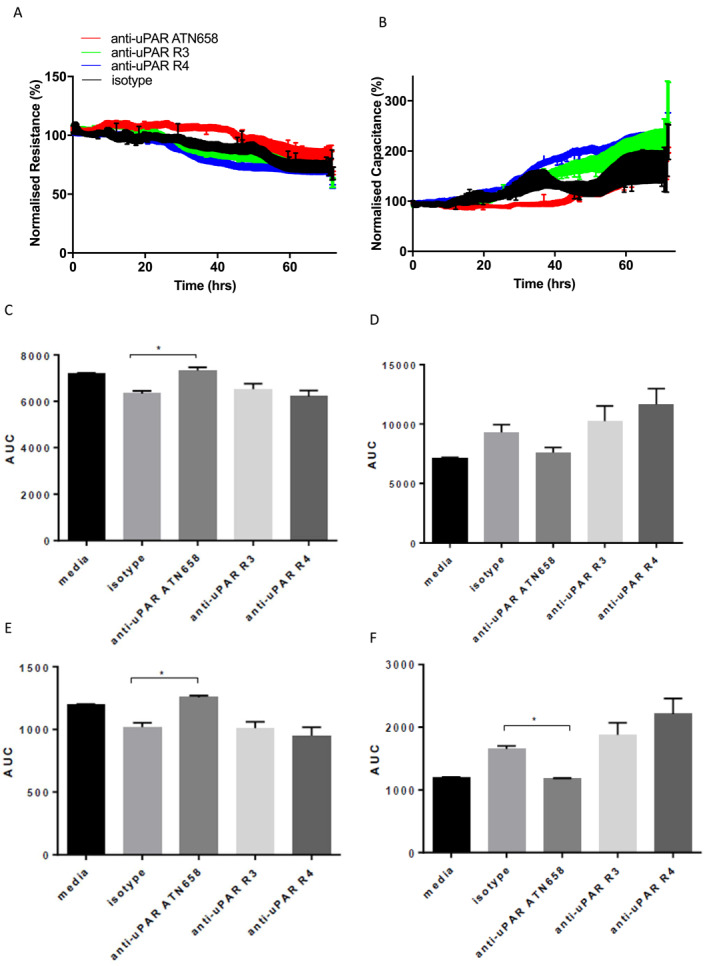
Treatment with anti‐uPAR ATN‐658 improves bronchial epithelial barrier function and alters cell adhesion. Bronchial epithelial cells from control subjects, grown in submerged cultures were treated with several urokinase plasminogen activator receptor (uPAR) blocking antibodies; anti‐uPAR R3, anti‐uPAR R4 that target the uPA‐uPAR interaction and anti‐uPAR ATN‐658, that targets uPAR‐integrin interactions or IgG1 isotype control (1 mg/mL). Real‐time Electrical cell impedance sensing (ECIS) traces for (A) resistance (barrier) and (B) capacitance (adhesion/spreading) of cultured cells. Resistance (Ω) and capacitance (C), were monitored at 400 Hz and 32 KHz, respectively, for 72 h. C/D and area under the curve (AUC) analysis was performed using resistance and capacitance measurements taken from 0 to 72 h post treatment, showing ATN‐658 had a significant effect on resistance. The changes in membrane properties were greatest at 36–48 h (E/F), with ATN‐58 treated cells showing increased resistance and decreased capacitance. Data represent mean ± SEM (*n* = 4).

Having identified that scuPAR negatively correlates with epithelial barrier integrity at ALI and in submerged, undifferentiated cultures, we next explored whether we could selectively manipulate uPAR function to improve the barrier. We used a series of anti‐uPAR monoclonal antibodies to selectively block specific uPAR protein epitopes, that is, anti‐uPAR R3 (which binds to domain 1) and R4 (which binds to domain 3), both of which block uPA‐uPAR interactions, and anti‐uPAR ATN‐658, which blocks uPAR‐integrin interactions with barrier function and adhesion. Treatment with ATN‐658, in pre‐clinical development for cancer,^20^ improved cellular resistance (barrier) and reduced capacitance (adhesion/spreading) when compared to the isotype control in 36–48 h post‐treatment, but not at earlier or later time‐points (Figure 4A–F). Targeting the uPA binding sites on uPAR using R3 and R4 monoclonal antibodies did not result in a significant change in resistance or capacitance. These data provide initial support that blocking specific functions of uPAR can improve epithelial barrier properties, albeit in vitro and to a modest magnitude of effect (20%–28%).

### 3.5 Transcriptomic analyses to provide insight into the role of selective uPAR isoforms

**FIGURE 5:**
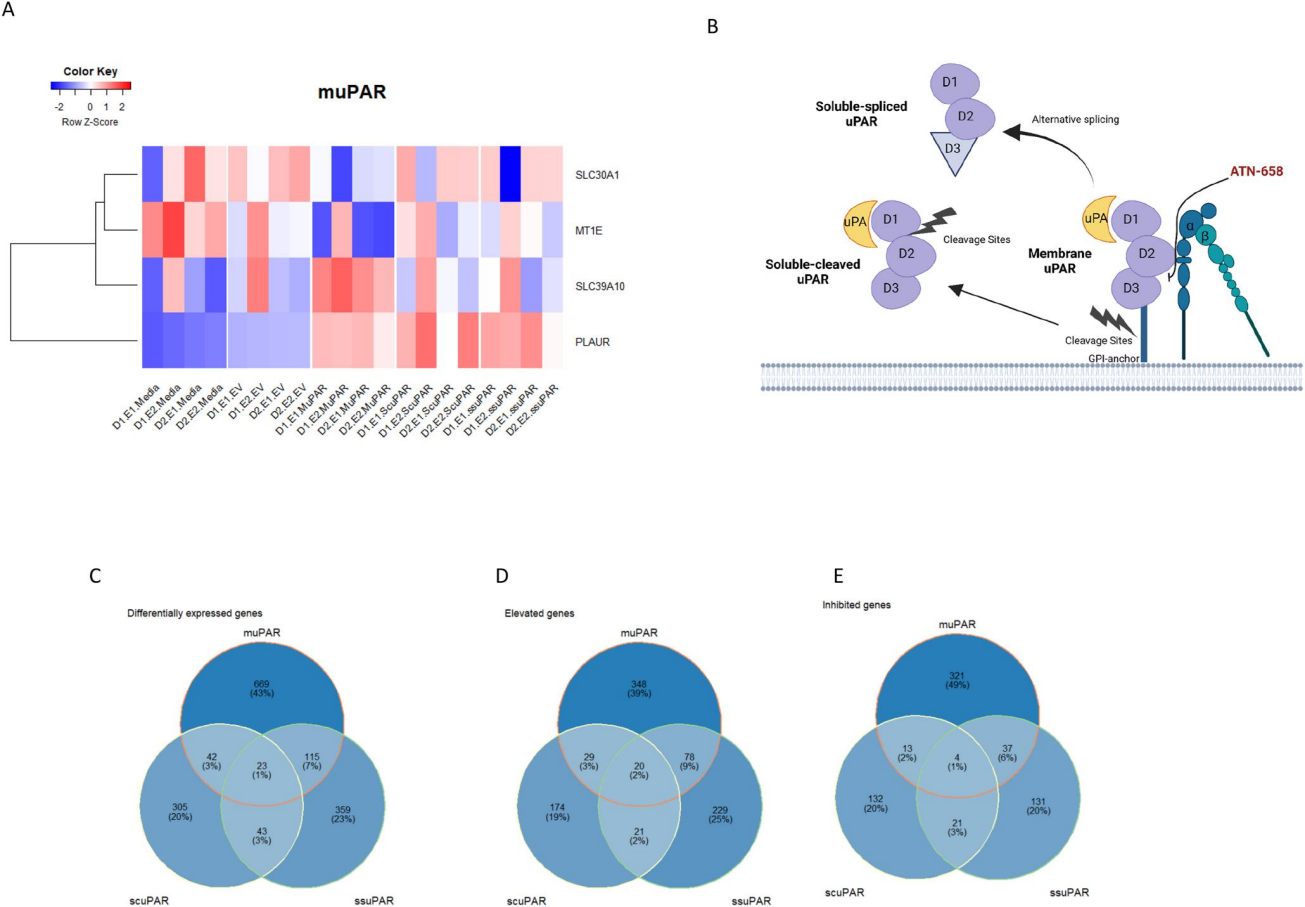
Whole transcriptome analysis identifies that urokinase plasminogen activator receptor (uPAR) protein variants have different and overlapping effects on bronchial epithelial cell gene expression. Bronchial epithelial cells were engineered to over‐express muPAR, scuPAR, or ssuPAR. A HeatMap for the genes with the greatest fold change identified in cells overexpressing membrane uPAR (Panel A) identifies changes in MT1E, SLC30A1 and SLC39A10 unique to the muPAR overexpressing population when compared to empty vector (pcDNA3) control (*Q*‐value ≤0.05). PLAUR was shown to be overexpressed in all overexpression cell models (*Q*‐value ≤0.05). (B) A diagrammatic illustration of how uPAR protein variants are generated. (C–E) Overlap in genes identified as differentially expressed in response to over expression of the different isoforms at a nominal *p* < 0.05 identified that 23 genes including *PLAUR* were differentially expressed following elevated expression of all three uPAR isoforms, all presenting the same directionality of effect (Panels C‐E).

We next set out to further our initial experiments that identified elevated expression of total uPAR in bronchial epithelial cells isolated from asthma patients and highlighted potential functional roles for the different forms of the receptor, that is, the relationship between suPAR and barrier formation, increased proliferation, decreased migration, and increased plasmin and total MMP‐9 expression.^14^ We set out to provide a greater understanding of the functional role of uPAR isoforms in epithelial cell biology using a gain of function approach combined with transcriptomics. More specifically, we engineered primary bronchial epithelial cells to over‐express ssuPAR, a recombinant scuPAR isoform, mimicking the proteolytic release of uPAR from the cell or the muPAR isoform, and then completed RNA‐seq analyses (Figure 5). All RNA used for RNA‐seq had a RIN score >9. Samples presented with 95% mapped reads and an average of 54,463,897 reads (41,336,063‐60,746,928).

The minimal differential gene expression was observed following muPAR overexpression (Table 1, Figure 5A; *MT1E*, *SLC39A10* & *SLC30A1*; *q*‐value ≤0.05). Reassuringly, uPAR (*PLAUR* mRNA) was also elevated (*p* = 6.9 × 10^−3^, logFC = 3.07). No statistically significant transcriptomic changes were observed following scuPAR or ssuPAR overexpression; however, *PLAUR* was still seen to be overexpressed in these experiments (scuPAR: *Q* = 0.01, logFC = 3.88; ssuPAR: *Q* = 0.03, logFC = 3.16). While not meeting stringent significance (*q*‐value ≤0.05) we also compared the overlap in genes identified as differentially expressed in response to over expression of the different isoforms at a nominal *p* < 0.05 (Figure 5C,E, Tables S2A–C). These data highlighted that 22 genes, excluding *PLAUR*, were differentially expressed following elevated expression of all three uPAR isoforms, all presenting the same directionality of effect (3 decreased, 19 elevated, see Table S2D). Interestingly 181 genes were differentially expressed, and presented with the same directionality, following over‐expression of two or more uPAR isoforms (Table S2E). The elevated genes include those associated with cellular proliferation (*BRICD5*), those previously associated with asthma and other inflammatory diseases (*IL1RL1*, *IL6R*) and those involved in inflammatory cell signaling (*LIME1*) and function (*SLC11A1*). All comparisons were made to cells transiently transfected with an empty vector‐negative control.

**TABLE 1:** Genes and regulatory RNAs differentially expressed following elevated expression of membrane urokinase plasminogen activator receptor.

Gene	Gene name	Mean expression	B	logFC	Q.Val	Known function
*MT1E*	Metallothionein 1E	6.01984	10.20492	−1.65	0.000134	Predicted to enable zinc ion binding activity. Involved in cellular response to cadmium ion and cellular response to zinc ion.
*SLC39A10*	Solute carrier family 39 member 10	5.313369	8.187041	1.278023	0.000649	Metal ion SLC transporters and transport of inorganic cations/anions and amino acids/oligopeptides.
*PLAUR*	Urokinase plasminogen activator receptor	9.531773	5.624503	3.067701	0.006909	Target receptor for over‐expression
*SLC30A1*	Solute carrier family 30 member 1	6.087036	4.167797	−0.58896	0.023875	Metal ion SLC transporters and transport of inorganic cations/anions and amino acids/oligopeptides.

*Note*: Human bronchial epithelial cells (*n* = 4) were engineered to overexpress muPAR, whole transcriptome analysis was performed to identify muPAR function. Differential gene expression analysis identified the log2 fold change and adjusted *p*‐value (*q*‐value) for genes measured and identified 4 DE genes (FDR <10%) compared to the empty vector control.

## 4 DISCUSSION

In this study, we set out to expand our understanding of the role of uPAR in bronchial epithelial cell function in the context of asthma. Urokinase plasminogen activator receptor may represent a therapeutic opportunity for asthma if selective functions of uPAR can be identified and targeted while maintaining essential functions, for example, fibrinolysis. We demonstrate that (i) elevated uPAR expression is a feature of cultured asthma HBECs, suggesting that the previously observed elevated expression in vivo is intrinsic to asthma patient cells and (ii) scuPAR expression is variable during bronchial epithelial differentiation in vitro and is inversely correlated with barrier properties in ALI and submerged culture conditions. We identified that targeting uPAR using a selective blocking antibody targeting uPAR‐integrin binding can enhance these barrier properties. This is suggestive of a therapeutic potential in asthma, where the epithelial barrier is compromised. Finally, using a gain of function approach, we did not identify a statistically significant effect on the transcriptome in cells over‐expressing uPAR, suggesting that receptor activation is needed to identify isoform‐driven functional effects. Using a nominal cut off of *p* ≤ 0.05 we did however identify a uPAR signature, including an overlap of 22 differentially expressed genes universal to all three uPAR sub‐types, including key genes in *BRICD5*, *IL1RL1*, *IL6R*, *LIME1* and *SLC11A1* (Table S2D, Figure 5C–E). These data provide a novel insight into the role of uPAR in asthma and suggest that selectively targeting uPAR may be feasible to improve the airway barrier properties known to be defective in asthma.

We and others have shown that uPAR expression is elevated in the airways of people with asthma^4^, ^7^ and that this expression and activity drives downstream mediators relevant to airway remodeling, for example, MMPs.^4^ Similarly, elevated scuPAR has been observed in the sputum of asthma and COPD patients and correlated with lung function,^9^ with elevated scuPAR present in the serum of severe non‐allergic asthma patients.^13^ More recently, elevated serum suPAR levels have been associated with increased rates of hospital re‐admission and mortality in asthma patients.^22^ These findings provide support for the functional role of uPAR in asthma. Airway structural cell changes were implicated in our genetic association studies showing that genetic polymorphisms spanning *PLAUR* were associated with (i) increased risk of developing asthma, (ii) bronchial hyperresponsiveness, and (iii) accelerated lung function decline in patients.^6^ We subsequently identified that uPAR airway epithelial in vivo expression correlated with epithelial cell proliferation, with genetic variants driving this correlation, at least in part.^8^ Interestingly, uPAR expression in the airway epithelium in COPD has been shown to be correlated with lung function.^23^ These data suggest an altered uPAR expression in the airway epithelium in multiple obstructive lung diseases, including asthma. However, the fundamental question, “are these expression differences intrinsic to the airway epithelium of patients or induced by the lung environment in vivo?” remained. The current study demonstrates that the total uPAR mRNA expression is significantly elevated in bronchial epithelial cells isolated from asthma patients and cultured in vitro, that is, devoid of the stimulation/inflammatory environment. This suggests an intrinsic elevation of uPAR and potential gain of function in asthma patient cells.

A functional role for uPAR in epithelial cell homeostasis has been described, especially epithelial‐mesenchymal transition, proliferation and migration, influencing wound repair mechanisms.^4^, ^24^, ^25^, ^26^, ^27^, ^28^ However, the contribution of uPAR to bronchial epithelial cell barrier formation, which is known to be altered in asthma, remains to be determined. In this study, we demonstrate that uPAR expression changes over the course of epithelial cell differentiation, where an in vitro barrier is formed, peaking at 14 days. We are the first to show that uPAR expression is negatively correlated with the barrier properties of the developing cell layer. Interestingly, levels of uPA and MMP‐9 were negatively correlated with barrier properties, suggesting that alterations of the uPA‐uPAR‐MMP‐9 pathway mediate this observation; however, it is important to note that we did not demonstrate this directly. uPA‐uPAR pathway interaction leads to activation of pro‐remodeling factors, for example, TGFβ1 and MMP‐9, downstream of the protease cascade.^1^, ^4^ The association between uPAR and MMP‐9 activation is important as MMP‐9 has been directly implicated with airway remodeling^29^ and reduced HBEC‐barrier integrity.^30^ In a 2D model, uPAR levels positively correlated with adhesion/spreading as previously reported by the cancer field,^31^, ^32^ which may in part explain the relationship between uPAR and epithelial repair/regeneration mechanisms, which contribute to defective barrier formation in asthma.^33^ Crucially, this suggests that correctly targeting uPAR could enhance the epithelial barrier. Interestingly, recently in the context of IBD, Caco‐2 cells deficient in uPAR were protected from cytokine‐induced damage to the barrier (TEER) confirming the role of this receptor in epithelial cell‐barrier homeostasis,^34^ while in PLAUR deficient mouse models uPAR‐inducible pathways were shown to be necessary for the remodeling of the epithelial (podocyte) barrier in the kidney.^35^

We have previously demonstrated that blocking uPA‐uPAR binding attenuated the repair of damaged airway epithelium.^4^ Interestingly, blocking uPA‐uPAR binding in cancer cell lines inhibited invasion but had little effect on migration and no effect on adhesion, suggesting a role for other binding partners.^36^ To determine the binding partners/mechanisms underlying the relationship between uPAR expression and epithelial barrier formation, we used a series of selective anti‐uPAR antibodies that block epitopes known to interact with specific binding partners, for example, uPA, integrins. These data reveal that it was only the anti‐uPAR antibody (ATN‐658) that promoted an improvement in barrier properties and thus provide additional support that uPAR regulates airway epithelial permeability/barrier properties. Interestingly, ATN‐658 selectively blocks the phosphorylation of Akt, FAK and p44/42 MAPK in cancer cells.^37^ The inhibition of these pathways has previously been implicated in improving the airway epithelial barrier, for example, PI3K,^38^ p44/42 MAPK.^39^ ATN‐658 can inhibit growth, invasion, and metastasis of multiple carcinoma cell lines and has antitumor effects in vivo.^37^ The epitope for ATN‐658 on uPAR maps to amino acids 268–275 in Domain III and this antibody has been shown to block α5β1—fibronectin‐mediated cell adhesion.^20^ These data are in good agreement with our findings, identifying decreased adhesion/spreading of bronchial epithelial cells in the presence of ATN‐658, highlighting that uPAR functionality may be important for airway barrier formation and can be modulated. A complementary series of investigations demonstrated that targeting the uPA—uPAR interaction by either small molecules (IPR‐1110) or antibodies (ab5329) in Caco‐2 cells protected the epithelial barrier from cytokine induced damage.^34^ These data suggest different functions for uPAR, via different interactions, for maintaining a barrier versus repairing/protecting the barrier from insult.^34^

Urokinase plasminogen activator receptor is a complex receptor, with the overlapping and distinct functional role of different forms produced by splicing or protein cleavage not clearly defined. Evidence from the literature suggests that scuPAR is a chemoattractant, which is involved in the sequestration of uPA and is associated with hyperproliferation of HBEC.^4^, ^7^, ^14^ In HBECs, scratch wounding increases levels of scuPAR in supernatants and overexpression of membrane uPAR leads to increased receptor shedding and attenuation of scratch wound healing.^14^ Mechanical air/CO_2_ compression injury, mimicking bronchoconstriction, induces *PLAUR* and suPAR protein expression, linked to increased MMP‐2 and MMP‐9 activation.^7^ The generation of uPAR isoform gain of function cell lines identified specific cellular effects, affecting cellular effects such as proliferation, plasmin activity and MMP‐9.^14^

RNA sequencing, carried out to provide greater insight into these effects at the molecular level and identify novel isoform‐driven biology, determined that receptor isoform elevation had a limited overall effect on the epithelial transcriptome in the absence of activation. Of note, three genes were identified in the membrane uPAR over‐expression experiments meeting stringent statistical thresholds, namely *MT1E* (reduced), *SLC39A10* (elevated) and *SLC30A1* (reduced) in addition to *PLAUR* elevated) as expected. Metallothioneins (MT) are intracellular proteins involved in binding of metal ions and of interest as *MT1E* has been shown to be elevated in non‐small‐cell lung cancer tissue predicting outcome potentially by altering cell proliferation/apoptosis.^40^ Both SLC proteins have been linked to transport of metal ions and elevated *SLC39A10* was linked to poor prognosis in Hepatocellular Carcinoma^41^ via changes in proliferation, migration and apoptosis. *SLC30A1* has also been linked to the apoptotic response in the airway epithelium, albeit in the presence of viruses.^42^ Therefore, remarkably, all three genes identified are related to the binding of metals and regulating cell proliferation, migration and apoptosis, mechanisms linked to uPA/uPAR function also.

Using a nominal statistical threshold of *p* < 0.05, we identified a number of isoform specific genes (muPAR = 669; scuPAR = 305; ssuPAR = 359) and further 181 genes with an overlap across 2 of the uPAR isoforms (Table S2A–C,E). Additionally, uPAR overexpression universally caused changes in the expression of genes related to cellular proliferation (*BRICD5*) and T‐cell/B‐cell signaling (*LCK/LIME1*) and importantly asthma via the IL33 receptor (*IL1RL1*), now a target for new asthma drugs.

While we provide compelling data to suggest that uPAR expression is elevated in asthma bronchial epithelial cells, affecting epithelial barrier integrity with therapeutic targeting potential, it is important to recognise study limitations, for example, the small number of experimental donors and modest effect sizes. Similarly, the use of blocking antibodies has limitations including the potential for effecting neighboring domains of uPAR limiting precise mechanistic understanding and the often observed isotype effect as demonstrated in the current study. Therefore, our uPAR blocking experiments need additional validation using different approaches to confirm the potential to target this receptor to improve the epithelial barrier. Similarly, while we identify new potential functions of uPAR isoforms, these findings require replication and mechanistic and functional characterisation, particularly in the presence of uPAR activation.

Overall, this study provides a significant advance in our understanding of uPAR expression and function in the context of bronchial epithelial cells and asthma. Importantly, this insight and our proof of concept work suggests that selectively blocking specific integrin‐related functions of uPAR shows therapeutic potential for disorders characterized by a compromised epithelial barrier, for example, asthma.

## AUTHOR CONTRIBUTIONS

**Michael A. Portelli**: Conceptualization (supporting); data curation (equal); formal analysis (equal); funding acquisition (supporting); investigation (equal); methodology (equal); project administration (supporting); visualization (equal); writing—original draft (equal); writing—review & editing (lead). **Sangita Bhaker**: Data curation (equal); formal analysis (equal); investigation (equal); methodology (equal). **Vincent Pang**: Formal analysis (supporting); methodology (supporting); software (supporting); validation (supporting). **David O. Bates**: Methodology (supporting); resources (supporting); supervision (supporting). **Simon R. Johnson**: Resources (supporting). **Andrew P. Mazar**: Resources (equal); validation (equal). **Dominick Shaw**: Investigation (supporting); methodology (supporting); project administration (supporting); resources (equal); supervision (equal). **Christopher Brightling**: Methodology (supporting); resources (supporting). **Ian Sayers**: Conceptualization (lead); data curation (equal); formal analysis (equal); funding acquisition (lead); investigation (equal); methodology (equal); project administration (lead); resources (lead); supervision (lead); visualization (equal); writing—original draft (lead); writing—review & editing (supporting).

## CONFLICT OF INTEREST STATEMENT

Dr Michael Portelli, Dr Sangita Bhaker, Dr Vincent Pang, Prof David O. Bates, Prof Dominick Shaw & Prof Simon R. Johnson declare no conflict of interest. IS reports a collaboration grant from Boehringer Ingelheim related to this work and collaboration grants from GlaxoSmithKline (GSK) Funding and Biotechnology and Biological Sciences Research Council not related to this work. Prof. Andrew P. Mazar owns equity in Monopar Therapeutics, the company that owns the rights to ATN‐658. Andrew P. Mazar is also a co‐inventor on multiple patents that cover ATN‐658 and related molecule composition of matter.

## CAPSULE SUMMARY

This study identified that bronchial epithelial cells from asthma patients have elevated uPAR levels, that may in part contribute to a defective barrier and blocking specific uPAR functions can enhance this barrier, providing a therapeutic opportunity.

## Supporting information

Supporting Information S1

Table S2

## Data Availability

The data that support the findings of this study are available from the corresponding author upon reasonable request.
