# Supplementary material for: Elevated PLAUR is observed in the airway epithelium of asthma patients and blocking improves barrier integrity
Source: Clin Transl Allergy. 2023 Oct 1;13(10):e12293. doi: 10.1002/clt2.12293 (PMC10542610; doi:10.1002/clt2.12293)
Supplement: Supplementary file 1 — Supporting Information S1 [file CLT2-13-e12293-s001.docx]

## **Supplementary Materials**

**Novel insight into urokinase plasminogen activator receptor function in the airway epithelium in asthma and therapeutic targeting**

Sangita Bhaker, PhD^1*^, Michael A Portelli, PhD^1*^, Vincent Pang, PhD^2^, Prof. David Bates, PhD^2^, Prof. Simon Johnson, PhD^1^, Prof. Andrew P Mazar, PhD^3^, Prof. Dominick Shaw, PhD^1^, Prof. Christopher Brightling, PhD^4^ & Prof. Ian Sayers, PhD^1^.

*^1^Centre for Respiratory Research, NIHR Respiratory Biomedical Research Centre, Biodiscovery Institute, University Park, University of Nottingham, Nottingham, UK.*

*^2^Tumour Vascular Biology Group, Biodiscovery Institute, University Park, University of Nottingham, Nottingham, UK.*

*^3^Feinberg School of Medicine, Northwestern University, Evanston IL, USA.*

*^4^University of Leicester and Department of Respiratory Medicine University Hospitals of Leicester NHS Trust, Leicester, United Kingdom*

Corresponding author: Dr. Michael Portelli; Centre for Respiratory Research, NIHR Respiratory Biomedical Research Centre, School of Medicine, Biodiscovery Institute, Science Road, University Park, University of Nottingham, Nottingham, UK.

[michael.portelli@nottingham.ac.uk](mailto:michael.portelli@nottingham.ac.uk)

***Supplementary Methods***

***Immunofluorescence and immunohistochemistry of paraffin-embedded ALI sections***

Paraffin wax was removed from sections using Histoclear II. Sections were washed and dehydrated in 100% ethanol, re-hydrated in clean 95%v/v ethanol and rinsed in distilled water. Sections were immersed in warmed Sodium Citrate Buffer (pH 6) for 40 min and were washed with sterile water. Cells were permeabilised, washed and blocked (10%v/v Goat Serum in PBS) at room temperature for 20 min. Sections were then washed in sterile water and incubated with anti-uPAR antibody (Sigma, #SAB4200412) overnight at 4°C. Sections were then washed and incubated with secondary antibody for 1 h at room temperature. For Haemotoxylin and Eosin staining, sections were stained with Mayers Haematoxylin, washed and stained Eosin. Mucin detection was carried out by staining with Alcian Blue. Sections were then washed and mounted with DAPI (Vectamount, VectorLabs # H-5000).

***RNA-sequencing, differential gene expression and pathway based analyses***

Library preparation and sequencing was performed by Deep-Seq, University of Nottingham. Paired-end sequencing was performed using the Illumina NextSeq500 and generated 60 million paired end reads (75bp) per sample. Adapter and quality trimming of reads were performed using Trimmomatic[^18^](#_ENREF_18) (v0.39) and quality analysis carried out using FASTQC (v0.10.1) (Simon Andrews, Babraham Bioinformatics). Reads were aligned to human assembly GRCh38 using the STAR alignment tool[^19^](#_ENREF_19) (v27.10a). Differential gene expression analysis was determined using EdgeR and Glimma, following precision weighting using voom in order to generate linear-modelling strategies. Due to observed clustering of data based on epithelial cell donor, data was blocked (normalized) based on donor ID following mean-variance modelling of samples. Differential gene expression was determined via Limma using the ‘decideTests’ argument to summarise the results of the linear model tests. The adjusted p-value was determined using the Benjamini and Hochberg’s method to control the false discovery rate.

***Supplementary Figures***

**Fig. S1. scuPAR protein expression in supernatants modestly correlates with MMP-9, uPA and PAI-1 protein levels in cultured bronchial epithelial cells from asthma patients.** Supernatants from cultured HBEC brush samples from asthma and healthy individuals (passage 2-3) were collected and levels of scuPAR (soluble-cleaved uPAR), uPA (urokinase), MMP-9 (matrix metalloproteinase 9) and PAI-1 (plasminogen activator inhibitor 1) were determined by Luminex . scuPAR levels were positively correlated with MMP-9, uPA and PAI-1 in cells from asthma patients. Protein levels did not correlate in control subject cells.

1. b)


c) d)

**Fig S2.** **uPAR pathway component expression is not related to the barrier function of bronchial epithelial cells with an established barrier at Day 21.** Correlation between scuPAR, uPA, MMP-9 levels in basolateral supernatants of untreated NHBEC and TEER at day 21 ALI. a) r=-0.26, P=0.24, n=22, b) r=-0.57, P=0.15, n=8, c) r=-0.28, P=0.19, n=23, d) r=0.19, P=0.50, n=14. Data for NHBEC donor 3 only where n=individual data points and representative of other donors.

**A**


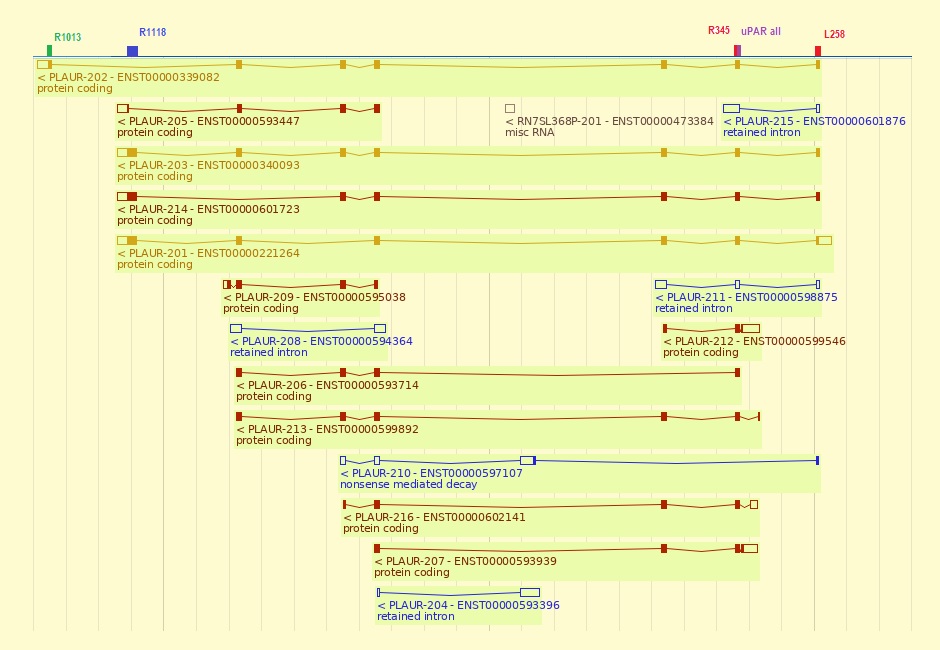


**B**

| Target | Forward Primer | Reverse Primer |
| --- | --- | --- |
| Total PLAUR | PLAUR E1 L258  CTGCTGCTGCTCCACACCT | PLAUR E2 R345  ACTCTTCCACACGGCAATCCC |
| Membrane PLAUR | PLAUR E1 L258  CTGCTGCTGCTCCACACCT | E7V1 R1118  TGGGTGGTTACAGCCACTTT |
| Soluble PLAUR | PLAUR E1 L258  CTGCTGCTGCTCCACACCT | suPAR R1013  TGGCAACCAGCTTCCCCAGAGT |
| Probe | uPAR all  CCAGCCTCTTGGGGCCTGCGGT |  |

**C**

| Primer | Sequence | Exon | Location (GRCh37) |
| --- | --- | --- | --- |
| PLAUR E1 L258 | CTGCTGCTGCTCCACACCT | 1 | 44174226-44174245 |
| PLAUR E2 R345 | ACTCTTCCACACGGCAATCCC | 2 | 44171775-44171796 |
| E7V1 R1118 | TGGGTGGTTACAGCCACTTT | 7A | 44153162-44153181 |
| suPAR R1013 | TGGCAACCAGCTTCCCCAGAGT | 7B | 44150672-44150694 |
| uPAR all | CCAGCCTCTTGGGGCCTGCGGT | 2 | 44171861-44171839 |

**Fig S3: Panel A identifies the physical location of primers and probes utilised for the determination PLAUR mRNA levels using Taqman qPCR (Panels B&C) relative to know PLAUR isoforms as presented on Ensemble (04.05.2023).**

|  | Asthma | Control |
| --- | --- | --- |
| Subject (n) | 33 | 18 |
| Age  Mean ±SD | 53 ±14 | 39 ±16 |
| Gender (% Female) | 61 | 25 |
| FEV_1_ (% predicted)  Mean ±SD | 79 ±17 | 101 ±10 |
| FEV_1_/FVC  Mean ±SD | 79 ±8 | 68 ±11 |
| BTS/GINA >3  (% moderate/severe asthma) | 91 | - |
| Atopy (% at least one positive indication) | 50 | 0 |
| Mean blood Eosinophils (x10^9^/L) Mean ±SD | 0.48 ±0.49 | 0.13 ±0.07 |
| Mean Serum igE (kU/L)  Mean ±SD | 620 ±1371 | 52 ±72 |

**Table S1: Demographics for Asthma and Control Subjects utilised for RNA-sequencing, who underwent bronchoscopy to isolate bronchial epithelial cells.**

**Table S2: This is available as a standalone Excel Sheet.**
